# Placing Leishmaniasis in the Limelight through the Communicable Disease Surveillance System: An Experience from Sri Lanka

**DOI:** 10.3390/pathogens11060680

**Published:** 2022-06-13

**Authors:** Sonali Dinushika Gunasekara, Hasara Nuwangi, Nuwan Darshana Wickramasinghe, Kosala Weerakoon, Helen P. Price, Lisa Dikomitis, Suneth Buddhika Agampodi

**Affiliations:** 1 Department of Community Medicine, Faculty of Medicine and Allied Sciences, Rajarata University of Sri Lanka, Saliyapura, Anuradhapura 50008, Sri Lanka; hasara.ph@med.rjt.ac.lk (H.N.); nuwick@med.rjt.ac.lk (N.D.W.); suneth@med.rjt.ac.lk (S.B.A.); 2 Department of Parasitology, Faculty of Medicine and Allied Sciences, Rajarata University of Sri Lanka, Saliyapura, Anuradhapura 50008, Sri Lanka; kosalagadw@med.rjt.ac.lk; 3 School of Life Sciences, Keele University, Newcastle-under-Lyme ST5 5BG, UK; h.price@keele.ac.uk; 4 Kent and Medway Medical School, University of Kent and Canterbury Christ Church University, Canterbury CT2 7FS, UK; lisa.dikomitis@kmms.ac.uk

**Keywords:** leishmaniasis, disease surveillance, Sri Lanka

## Abstract

Having an effective surveillance system is imperative to take timely and appropriate actions for disease control and prevention. In Sri Lanka, leishmaniasis was declared as a notifiable disease in 2008. This paper presents a comprehensive compilation of the up-to-date documents on the communicable disease and leishmaniasis surveillance in Sri Lanka in order to describe the importance of the existing leishmaniasis surveillance system and to identify gaps that need to be addressed. The documents perused included circulars, reports, manuals, guidelines, ordinances, presentations, and published articles. The disease trends reported were linked to important landmarks in leishmaniasis surveillance. The findings suggest that there is a well-established surveillance system in Sri Lanka having a massive impact on increased case detection, resulting in im-proved attention on leishmaniasis. However, the system is not without its short comings and there is room for further improvements.

## 1. Introduction

Disease surveillance systems are important in the efforts to strengthen the prevention and control of spread of diseases. Effective surveillance systems provide essential information to take timely and appropriate actions for disease control and prevention, and to manage public health-care policies and programs [1]. Leishmaniasis was declared as a notifiable disease in Sri Lanka in 2008 and annually an increasing number of cases have been reported from many districts in the country [2]. In this paper, we present a comprehensive compilation and critique of the disease notification pathway for leishmaniasis and the current status of leishmaniasis reporting in Sri Lanka, while discussing how the routine communicable disease surveillance system has assisted in the increased case detection of leishmaniasis in the country.

### 1.1. Historical Background of Surveillance in Sri Lanka

Surveillance of communicable diseases includes the systematic collection, analysis, interpretation, and dissemination of data in order to facilitate and monitor the control and prevention of diseases. In Sri Lanka, notification of communicable diseases has been a legal requirement since 1897 under the Quarantine and Prevention of Diseases Ordinance [3]. All the medical practitioners attending to any of the notifiable diseases listed in Table 1 should report suspected or confirmed cases to the proper authority. Any person who breaches this regulation will be found guilty of an offence under the quarantine and prevention of diseases ordinance and shall be prosecuted in a magistrate court under Section 4 of the ordinance [3].

The sanitary branch of the civil medical department was established in Sri Lanka in 1915 and the main responsibility of this branch was on environmental sanitation and communicable disease prevention [4]. With the establishment of this department, six ‘sanitary inspectors’ were appointed following a six-month training period at the Ceylon Medical College. This category of healthcare professionals was mainly involved in the control and prevention of communicable diseases at that time [5]. Over time, the designation of ‘sanitary inspector’ has changed, and the designation was renamed as ‘Public Health Inspector’ (PHI) in 1954 [5].

One of the critical landmarks in the public health system in Sri Lanka was the establishment of the first ‘Health Unit’ in Kalutara, Western province in Sri Lanka in 1926 [4]. This unit introduced the concept of community-level preventive service delivery via a medical officer and a team of community-based healthcare professionals. During the formative years of the health unit system, the primary attention was on the control of communicable diseases and environmental sanitation [5].

### 1.2. Current Reporting Pathways of Communicable Diseases

**Figure 1:**
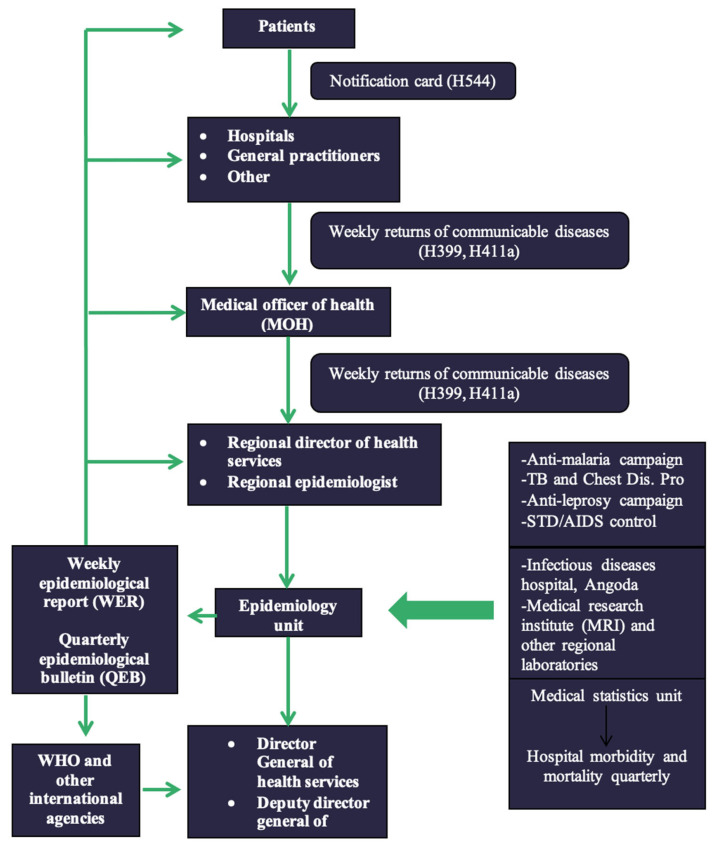
Communicable disease surveillance system in Sri Lanka.

A patient seeking medical care for communicable diseases is recognized and recorded by the Sri Lankan health system by two main routine reporting approaches:

- Surveillance of communicable diseases through the notifiable disease reporting system (Figure 1);
- Indoor morbidity and mortality reporting (IMMR).

- Surveillance of communicable diseases through the notifiable disease reporting system.

Surveillance of communicable diseases in Sri Lanka is mainly based on a system of notification of certain selected diseases. There are 28 communicable diseases on the current list of notifiable diseases (Table 1). Medical officers in all government and private medical institutions, all medical practitioners or registered healthcare persons professing to treat diseases and attend to patients, are responsible for notification. The national disease surveillance system of Sri Lanka consists of data collection, compilation, analysis, interpretation for action, and feedback. This system was built to identify the specific causes of morbidity and mortality to reduce the incidence of diseases [6].

**Figure 2:**
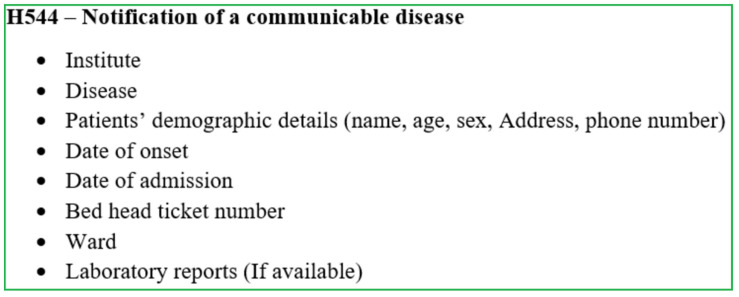
Components of Form H544—Notification of a communicable disease in Sri Lanka.

**Figure 3:**
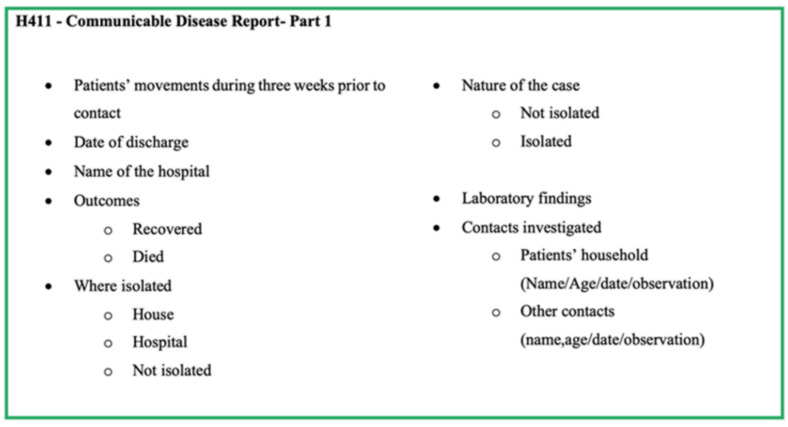
Components of form H411—Communicable Disease Report-Part 1 of the communicable disease surveillance system in Sri Lanka.

Generally, any medical practitioner treating a patient with a disease listed under the category of notifiable diseases should officially report the case through the local Medical Officer of Health (MOH) on clinical suspicion without waiting for the confirmatory laboratory investigations, using the ‘notification of a communicable disease’ form (H544) (Figure 2). Details provided in the H544 are entered in the ward and hospital notification registers before being forwarded to the MOH of the area for investigation. Once the completed H544 is received, the MOH records the details in the MOH notification register and refers to the relevant area PHI for investigation and confirmation. The PHI investigates the case and takes the necessary measures for disease control in the field, and hands over the communicable disease report-part I (Health 411 form—H411; Figure 3) along with H544 (Figure 2) to the MOH office within 7 days of the initial receipt of the H544.

**Figure 4:**
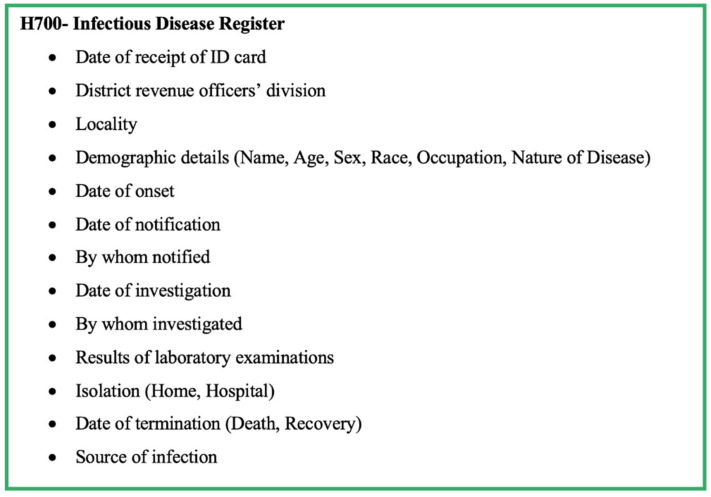
Components of form H700—Infectious Disease Register of the communicable disease surveillance system in Sri Lanka.

**Figure 5:**
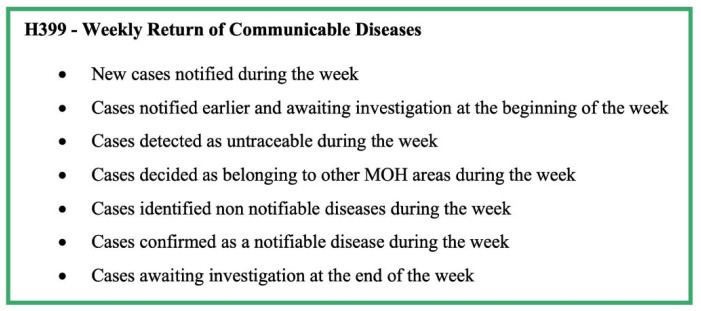
Components of form H399—Weekly Return of Communicable Diseases of the communicable disease surveillance system in Sri Lanka.

**Figure 6:**
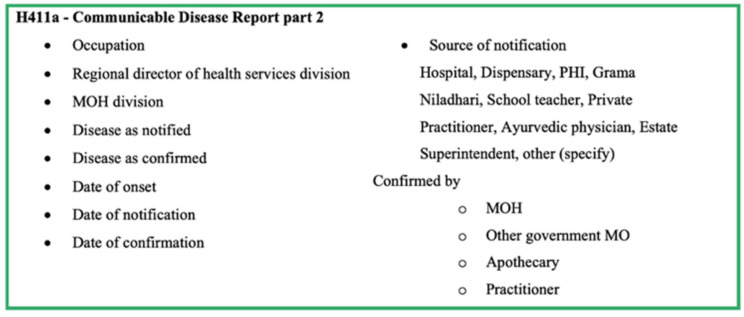
Components of form H411a—Communicable Disease Report-Part 2 of the communicable disease surveillance system in Sri Lanka.

Details on the H411 forms of confirmed cases are recorded in the Infectious Diseases Register (Health 700 form—H700; Figure 4). The MOH consolidates the weekly data in the Infectious Disease Register every Friday and prepares the weekly return of communicable diseases (WRCD—Health 399 form; Figure 5) and sends this to the Regional Director of Health Services (RDHS), Regional Epidemiologist (RE), and the chief epidemiologist at the Epidemiology Unit, on Saturday, with detailed information on the confirmed cases using the Infectious Disease Report—part II (Health 411a form—H411a; Figure 6). Further, the MOH updates the spot map and charts, which indicate the number of cases for each communicable disease in the area, and these are maintained at the MOH office for information and necessary actions at the local level.

The epidemiology unit of Sri Lanka (http://www.epid.gov.lk, accessed on 21 October 2021) is the apex body responsible for designing, implementation and monitoring of communicable disease surveillance. The epidemiology unit receives the H399 and H411a forms weekly through RDHS/RE and other special institutions such as the infectious diseases hospital at Angoda in the Western Province, Medical Research Institute (MRI) and other regional laboratories, the registrar general, anti-malaria campaign, tuberculosis, and chest disease program, anti-leprosy campaign, and sexually transmitted diseases/AIDS control, and quarterly updates form hospital morbidity and mortality through the medical statistics unit. Then these data are collected and analyzed by the epidemiology unit and sent to the Director General of Health Services and Deputy Director General of Health Services. Moreover, these data are published as weekly and quarterly epidemiological reports and sent back to the RDHS, Regional Epidemiologists (RE), MOH, and hospitals as well as to the WHO and other international agencies.

The numbers of cases of all notifiable diseases in Sri Lanka are published weekly in the Epidemiological Report [7] and quarterly in the Epidemiological Bulletin of the Epidemiology unit of Sri Lanka [8] and the public and any interested party can access those through the unit’s website.

**Figure 7:**
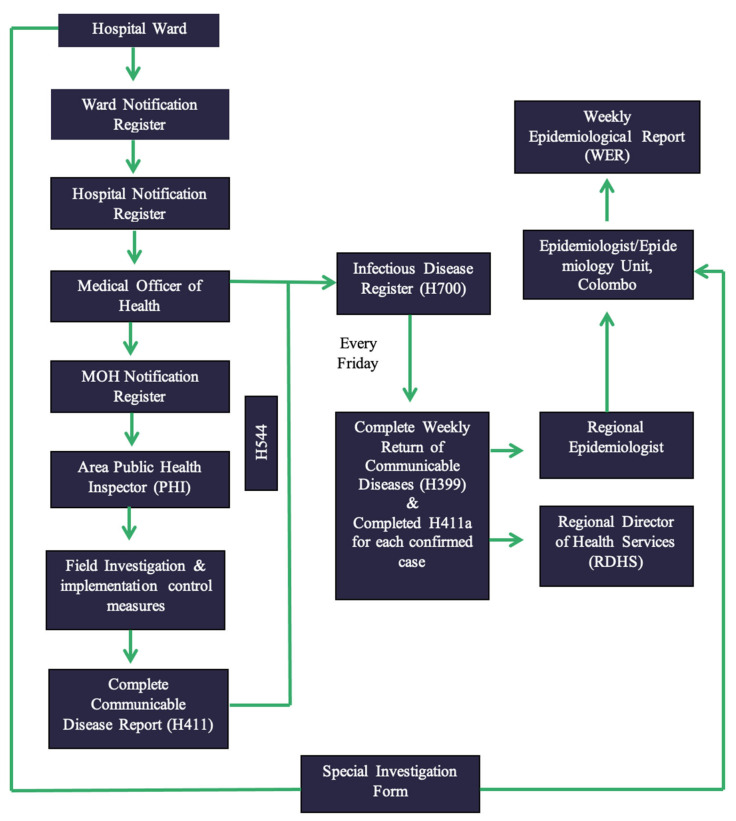
Flow chart of notifiable disease reporting and dissemination of information.

The disease reporting, feedback, and dissemination systems are outlined in Figure 7. In Sri Lanka, the allopathic medical system is primarily followed for treating notifiable diseases and there is no formal system in place to notify/record patients presenting only to ayurvedic or any other traditional medical practitioners [4].

- Indoor morbidity and mortality reporting (IMMR)

IMMR is the process of reporting the final clinical diagnosis of hospitalized patients based on the 10th revision of the International Classification of Diseases (ICD—10th version). Once an inward patient is discharged from a government hospital, the patient’s Bed Head Ticket (BHT) is sent to the medical records office at the hospital where all the records are archived. As a part of record keeping, the final diagnosis (in line with the international classification of diseases) mentioned in the BHT is entered in the indoor morbidity mortality register (IMMR). This information is used to formulate the morbidity and mortality quarterly return and subsequently the morbidity and mortality statistics in government hospitals. Cutaneous leishmaniasis is not separately identified in IMMR, and instead, it is recorded under the IMMR code 049 (International classification of diseases (ICD) code B55—Leishmaniasis, https://icd.codes/icd10cm/B55, accessed on 21 October 2021) with other infectious and parasitic diseases.

## 2. Results

### 2.1. Notification of Leishmaniasis

Leishmaniasis has been a notifiable disease in Sri Lanka since 2008. Any medical practitioner treating a patient with leishmaniasis should initiate the reporting process. For example, notification of leishmaniasis in the hospital should be made by the House Officer in charge of the ward, with a clinical diagnosis.

The H544 form is used to notify a communicable disease by government hospitals and forwarded to the MOH. The details are collected through the H544 form are shown in Figure 2. Apart from the basic demographic details and details from H544, the H411 (Communicable disease report part 1) and H411a (Communicable disease report part 2) forms report the information shown in Figure 3 and Figure 4. The H399 form gives a summary of the notifiable disease situation on a given week of a given MOH area (Figure 5).

**Figure 8:**
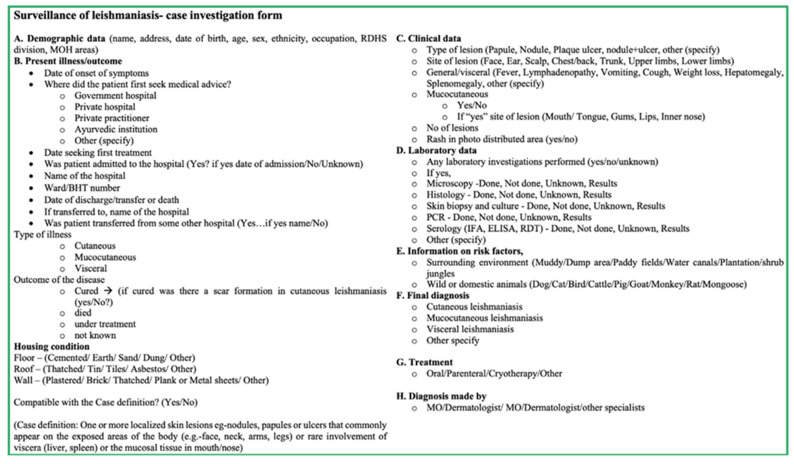
Special investigation form for leishmaniasis.

Reporting of leishmaniasis requires a special case investigation form (Figure 8) issued by the Epidemiology Unit of the Ministry of Health of Sri Lanka. It is advised that this investigation is carried out by a designated person personally. He/she should obtain the necessary details from the hospital or diagnosis card and additional information from the patient and relatives. Early investigation and return are deemed essential.

According to the current knowledge, many people including researchers believe that the first CL patient in Sri Lanka was recorded in the 1990s. However, evidence suggests that cases of leishmaniasis were detected in Sri Lanka earlier than the 1990s (manuscript under preparation). The first ever case with mucosal involvement was reported in 2005 [9]. In the same year, another case of MCL was reported with mucosal and nasal septum lesions [9,10] followed by the first case of VL in 2007 from Sri Lanka [11]. Annually, the number of reported CL cases has been increasing since this time. Cases have been reported from many districts in the country mainly from Anuradhapura, Polonnaruwa, Hambantota, Kurunegala, and Matara [2]. According to reports in 2019 alone, 3271 cases were reported across the country [7].

In 2012, the Sri Lanka College of Dermatologists issued a comprehensive set of guidelines on the management of leishmaniasis [12]. The guidelines consist of sections on diagnostic methods, which explains the clinical criteria for the diagnosis of cutaneous leishmaniasis and confirmatory tests. There are also detailed descriptions on available treatment options and instructions on how to administer each option together with a health education section on leishmaniasis explaining what the disease is, the importance of treatments, prevention, protective measures, and surveillance.

Under the section on surveillance the document states:

- “If a suspected case of leishmaniasis is found, it should be notified to the MOH of the residing area of the patient by using the health notification form (H544)
- Once the MOH receives the notification PHI will visit the patient’s residence and investigate within 7 days
- For every clinically confirmed case of leishmaniasis MOH team is supposed to fill out the special investigation form and send it to the epidemiology unit”.

In addition, the Sri Lanka government issued guidelines on the prevention and control of leishmaniasis in 2019 [13]. Guidelines include information on what should be collected on a number of different levels together with key messages to be given during field investigations/community awareness programs, at all healthcare institutional levels all medical officers of health levels, if the area is endemic for leishmaniasis, at the district level, and at the central level. The guidance is as follows:

#### 2.1.1. Healthcare Institutional Level

1. All suspected cases of CL and MCL should be referred to a dermatologist/dermatology clinic for early diagnosis and management;
2. All suspected cases of VL should be referred to a physician/pediatrician for further investigation and management;
3. All suspected/confirmed cases of leishmaniasis should be notified to the Medical Officer of Health in the patient’s area of residence at the earliest opportunity (H544, Figure 2);
4. All clinically or laboratory-confirmed leishmaniasis patients should be treated adequately and free of charge;
5. Patients should be followed up until complete cure and educated on the importance of continuing treatment;
6. The patient should be made aware of the disease and preventive measures.

#### 2.1.2. Medical Officer of Health Level

1. Every notified case should be entered in the MOH Office Communicable Disease Notification Register and field investigations should be completed within seven (7) days of the receipt of notification;
2. During the field investigation, history of similar illness, any history of interruption of treatment and reasons, close contacts with a similar lesion, occupational/travel history, and favorable environmental conditions for disease transmission should be investigated;
3. If PHI identifies any favorable conditions for disease transmission during their field investigations, advice should be provided by them to prevent the spreading of the disease in the area;
4. A follow-up plan for each patient should be prepared and implemented by the PHI;
5. All clinically/laboratory-confirmed cases should be entered into the Infectious Diseases Register (H 700) and sent to the Epidemiology Unit (Communicable Disease Part II: H–411a);
6. Screening for symptoms should be completed for all household contacts of confirmed leishmaniasis patients.

#### 2.1.3. If the Area Is Endemic to Leishmaniasis

1. Cases should be discussed at the monthly MOH conference;
2. A spot map should be maintained at MOH/PHI office;
3. If case clustering is observed:
  - active surveillance in the area should be organized by the MOH to identify and early referral of suspected leishmaniasis cases;
  - Entomological surveillance and a review of vector control approaches should be carried out;
  - After careful evaluation of entomological findings, the decision should be taken on indoor residual spraying with caution. No evidence has yet been generated on outdoor fogging as a preventative measure for leishmaniasis

#### 2.1.4. District Level

1. MOH—All suspected cases of leishmaniasis are notified from health institutions in the area;
2. Epidemiology Unit—All notified cases are investigated, followed up, and informed;
3. Regional Epidemiologist—All information on leishmaniasis is received on a regular basis;

#### 2.1.5. Central Level

The Epidemiology Unit should ensure the timely and complete receipt of all Weekly Returns of form H399—Communicable Diseases Returns. The Unit should analyze all the special surveillance forms of confirmed leishmaniasis cases, predict and intervene early in disease outbreaks, and review and discuss information on leishmaniasis during relevant forums. In addition to the above routine surveillance, special investigations are carried out for leishmaniasis, mainly through gathering additional information using a Special Investigation form (EPID/DS/LEISH/29/2010 form; Figure 8), which was introduced in 2010. The Special Investigation addresses patients’ clinical presentation, laboratory investigations, and clinical conclusions, which help detect confirmed cases out of the notified suspected cases. After the Special Investigation is completed, the form is sent back to the Epidemiology Unit, Colombo as soon as possible to update the central database.

### 2.2. Surveillance-Based Improved Responses to Leishmaniasis

**Figure 9:**
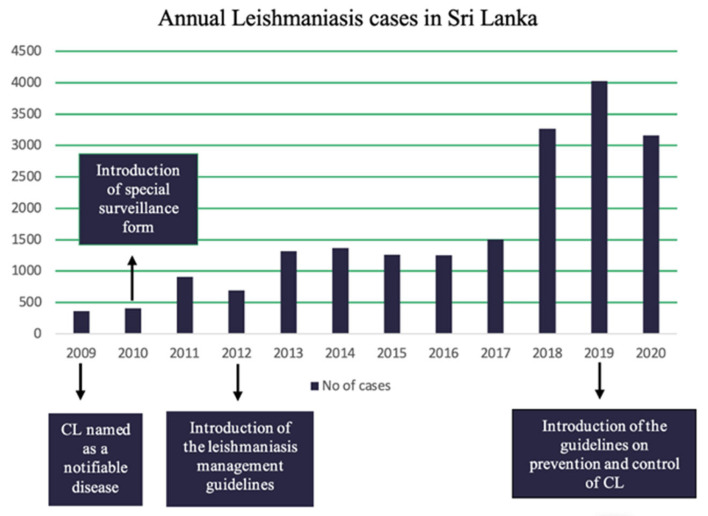
Annual leishmaniasis cases as reported through the routine surveillance system (data source: Epidemiology Unit).

Figure 9 indicates an increase in the number of annual cases of leishmaniasis in Sri Lanka. The highest number of annual cases, after the declaration of leishmaniasis as a notifiable disease were recorded in 2019. Introduction of the management guidelines and special surveillance for leishmaniasis has led to an increase in the number of reported cases in the subsequent years.

**Figure 10:**
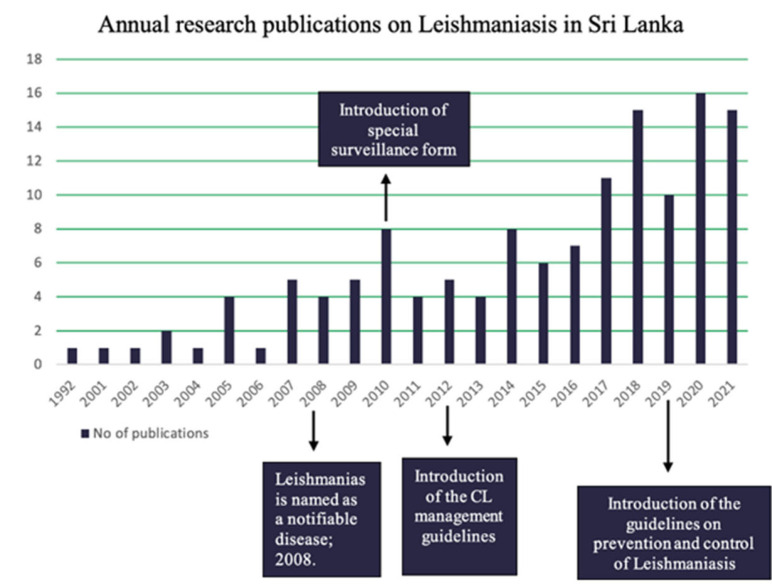
Annual research publications on leishmaniasis in Sri Lanka-PubMed.

Figure 10 indicates an increase in the number of annual research publications on leishmaniasis in Sri Lanka. Since the first publication on leishmaniasis in 1992, the highest number of publications was recorded in 2020.

## 3. Discussion

Sri Lanka has a well-developed surveillance system in place for communicable diseases, making the logistics of control and prevention of an emerging disease easier. When leishmaniasis was first made a notifiable disease in 2008, there was no need to introduce a new mechanism to report disease cases as a system was already in place. The only extra step that was introduced for the surveillance of leishmaniasis was a Special Investigation form. When duly completed, the Special Investigation form should provide all the data that are needed to assess and analyze the leishmaniasis situation in the country. However, in practice, whether this is being completed in every hospital or other health institution is questionable.

A study performed in the Mullaitivu district of Sri Lanka showed that the lack of a dermatologist and other resource issues such as lack of diagnostic facilities may disrupt the surveillance of the leishmaniasis [14]. This leads to underreporting and an interruption of the treatment process. As the surveillance system is an integral part of the improved disease response, this should be free of such pitfalls to achieve the end goal of control and prevention of the disease.

As shown in Figure 9 and Figure 10, the scientific evidence generated and case detection have increased after the milestone years of 2008, 2010, and 2012 as leishmaniasis was declared as a notifiable disease and CL management guidelines were introduced during this period. The guidelines issued in 2012 help the health practitioners to make a correct diagnosis, which may help to explain the increase in cases detected as shown in Figure 9. Since the guidelines have a separate section on surveillance, that could also have had a positive impact on case detection and reporting. In addition to the above possible explanations, the observed increase in the number of reported cases of leishmaniasis could also be due to an actual increase in the disease incidence. Nevertheless, this possibility needs to be explored further with meticulous analysis. While it is still too early to assess how the prevention and management guidelines released in 2019 will impact case detection, we believe it will result in increased case detection which will in turn lead to a better understanding of the disease, an improved response, and better treatment for people living with leishmaniasis. On the other hand, we may expect that there would be an actual reduction in the incidences of leishmaniasis due to the prevention and control activities implemented after the introduction of the 2019 guidelines. However, further research is needed to evaluate the impact of the recently introduced guidelines on the case detection and actual reduction in incidence.

The other routine system, IMMR, is not utilized in Sri Lanka with regards to leishmaniasis. We believe that it is now time to start using this well-established system for this disease. Now that leishmaniasis is an emerging condition in the country, it is necessary to prioritize this disease and report it separately instead of grouping it with other infectious and parasitic diseases in the IMMR. This will allow cross-validation of the reporting from the surveillance as part of the communicable diseases system which is important in assessing record accuracy. This in turn could be used to make better-informed decisions.

Furthermore, the existing surveillance system could easily be modified to generate better outcomes. One of the key missing elements from the case investigation form is the size of the wound. If this was added, it would be beneficial in establishing patterns and predicting future cases. Incorporating the cases of leishmaniasis reported from Siddha, Ayurveda, Unani, Acupuncture, and Homeopathy systems into routine notification and surveillance systems would also be beneficial in assessing the health-seeking behavior of patients and calculating the correct incidence of leishmaniasis in the country. For this purpose, the digitalization of the existing paper-based reporting system would be a practical and logical step. Training and educating the healthcare providers on WER and QEB and increasing the utilization to make informed decisions [15] is pivotal. Especially in regions where CL is highly prevalent, regional level data analysis and publications should be encouraged, thus making the surveillance data easily accessible. Steps should be taken to ensure that the reporting of leishmaniasis is not disrupted when other disease outbreaks occur in the country. We strongly recommend the digitalization of the data entries and real-time data access to utilize the system more efficiently.

To create a more improved response to the disease, we suggest that periodic inspections should be completed by the Epidemiology Unit of Sri Lanka to ensure that data are collected and reported routinely. In particular, completing the leishmaniasis Special Investigation form could be time-consuming, and when other public health matters arise this could be easily neglected. Furthermore, we strongly recommend making the data collected through the surveillance system available to interested third parties such as policymakers and researchers. We also recommend that future studies and reviews should be conducted on the practical issues of filling the Special Investigation form to improve the process.

The well-established surveillance system in Sri Lanka already has the potential to effectively identify and control possible outbreaks of newly emerging diseases. The existing system has already had a massive impact on increased case detection, resulting in improved attention on leishmaniasis which is considered an emerging disease. However, the system is not without its shortcomings and there is room for further improvements. We believe that implementing the recommendations discussed in this paper could make the system even stronger, which will then be used in the identification and control of newly emerging infectious diseases in the future.

## 4. Materials and Methods

We examined all publicly available documents relating to the communicable disease surveillance and leishmaniasis surveillance developed by the Ministry of Health, Sri Lanka, and especially the Epidemiology Unit in Sri Lanka. Reviewed documents included circulars, reports, manuals, guidelines, ordinances, presentations, and maps archived on the official websites of the Epidemiology Unit and the Ministry of Health, Sri Lanka. We manually collected the hard copies of the forms, records, and returns utilized in the routine communicable disease surveillance system in general and in leishmaniasis surveillance in Sri Lanka. We collected all those documents from the Teaching Hospital, Anuradhapura, and from the public health officials who are involved in the communicable disease surveillance including RE, MOH, and Supervising Public Health Inspectors (SPHI).

Since leishmaniasis was included as a notifiable disease in 2008, we compiled the data included in the Weekly Epidemiological Report (WER) and Quarterly Epidemiological Bulletin (QEB) issued by the Epidemiology unit, Sri Lanka, to report the annual number of leishmaniasis cases in Sri Lanka covering the period from 2008 to 2021. The disease trends reported were linked to important landmarks in leishmaniasis surveillance to describe the importance of the existing leishmaniasis surveillance system and to identify gaps that need to be addressed. In addition, we conducted a literature search on communicable disease surveillance and leishmaniasis surveillance in Sri Lanka using PubMed and Google Scholar to report the trend in the number of annual academic publications related to leishmaniasis in the country.

## Figures and Tables

**Table 1:** Current list of notifiable diseases in Sri Lanka.

**Group A**		
**Disease**	**Relevant Authority**	**Mode of Notification**
Cholera, Plague, Yellow Fever	Inform the Director General of Health Services, Deputy Director General (public health services I), the epidemiologist, regional epidemiologist, and medical officer of health immediately	by telephone, fax or email followed by form notification of a communicable disease (H544)
**Group B**		
Acute Poliomyelitis/ Acute Flaccid Paralysis, Measles, Rubella, Congenital Rubella Syndrome	The epidemiologist, regional epidemiologist, medical officer of health	by EPID/37/1/R2004-AFP/Suspected Poliomyelitis Form No.01 (Pink form) by telephone, fax, or email followed by form notification of a communicable disease (H544)
Chickenpox, Malaria, Meningitis, Mumps Dengue Fever, Neonatal Tetanus, Diphtheria, Dysentery, Encephalitis, Enteric Fever, Food Poisoning, Human Rabies, Leptospirosis, Leprosy, Leishmaniasis, Simple continued fever of over 7 days or more, Tetanus, Tuberculosis, Typhus Fever, Viral Hepatitis, Whooping Cough	The medical officer of health	by form notification of a communicable disease (H544)

## Data Availability

Not applicable.
